# Energy Efficiency Maximization for ME-IRS-Enabled Secure Communications

**DOI:** 10.3390/e28040432

**Published:** 2026-04-12

**Authors:** Chenxi Liu, Limeng Dong, Yong Li, Wei Cheng

**Affiliations:** School of Electronics and Information, Northwestern Polytechnical University, Xi’an 710049, China; liu_chenxi@mail.nwpu.edu.cn (C.L.);

**Keywords:** intelligent reflecting surface, secrecy energy efficiency, transmit beamforming, alternating optimization

## Abstract

This paper investigates the secrecy energy efficiency (SEE) maximization problem in a downlink multiple-input single-output (MISO) wireless communication system assisted by an intelligent reflecting surface with movable elements (ME-IRS). Unlike a conventional IRS, which has fixed-position elements, the proposed ME-IRS enables dynamic adjustment of element positions to exploit additional spatial degrees of freedom for performance enhancement. However, such flexibility introduces new challenges due to the strong coupling among transmit beamforming, IRS phase shifts, and element positions, as well as the additional power consumption caused by element movement. To address these issues, we formulate an SEE maximization problem by jointly optimizing the transmit beamforming, phase shift matrix, and element positions. The resulting problem is highly non-convex owing to the fractional objective function and coupled variables. To address this challenge, an efficient alternating optimization (AO) framework is developed by leveraging semidefinite relaxation (SDR), successive convex approximation (SCA), and gradient-based methods. Simulation results demonstrate that the proposed ME-IRS configuration significantly outperforms conventional fixed-position and discrete-position IRS configurations in terms of SEE, providing valuable insights into the impact of movable region size and system parameters.

## 1. Introduction

Intelligent reflecting surfaces (IRSs) have recently emerged as a promising paradigm for enhancing wireless communication performance by enabling adaptive reconfiguration of the radio propagation environment [1,2]. Specifically, by tuning the reflection coefficients of its passive elements, an IRS can facilitate constructive signal enhancement for legitimate users or destructive interference for unintended receivers, thereby improving both signal quality and secrecy performance [3,4]. Motivated by these advantages, extensive research efforts have been devoted to the joint optimization of transmit beamforming and IRS phase shifts [5,6,7,8].

In contrast to the aforementioned environment reconfiguration approach, movable antenna (MA) technology introduces a new degree of freedom by enabling position adaptability in wireless systems. Unlike conventional fixed-position antennas (FPAs), MAs can dynamically adjust their locations within a predefined region to better match the propagation conditions [9], which can be implemented via mechanically driven or electronically driven approaches [10]. By properly optimizing antenna positions, channel conditions can be significantly improved, leading to enhanced spectral efficiency, reliability, and secrecy performance [11,12]. Owing to this unique capability, MA has recently attracted growing attention in next-generation wireless communication research [13,14,15].

Although IRS and MA have been investigated as two independent techniques for enhancing wireless communications, it is natural to explore their integration to further improve system performance. Specifically, IRS focuses on reconfiguring the wireless propagation environment via phase adjustments, whereas MA introduces position adaptability at the transceiver side. By combining these two mechanisms, the system design can be extended beyond conventional phase-only or position-only optimization, enabling a more flexible manipulation of wireless channels. Recent work has begun to explore this integration. In ref. [16], a movable-element IRS (ME-IRS) architecture is proposed to mitigate the phase mismatch caused by discrete phase shifts and improve system performance. In ref. [17], an elementwise position optimization algorithm is developed to alleviate the local optimality issue encountered in conventional gradient-based methods. Moreover, movable elements have been incorporated into advanced frameworks such as simultaneously transmitting and reflecting (STAR)-RIS-assisted non-orthogonal multiple access (NOMA) and integrated sensing and communication (ISAC) systems to further enhance communication performance [18,19,20]. These studies demonstrate the potential of integrating element mobility into IRS design for performance enhancement.

Such enhanced flexibility is particularly beneficial for secure communications. Owing to the broadcast nature of wireless channels, confidential information transmitted over the air is inherently vulnerable to eavesdropping. In this context, physical layer security (PLS) has emerged as an effective approach to protect wireless transmissions without relying solely on cryptographic techniques. By properly designing the propagation environment and spatial signal distribution, both IRS and MA can be exploited to strengthen legitimate links while suppressing potential eavesdropping channels, thereby improving secrecy performance. For instance, movable-element-based STAR-RIS has been investigated for secure communication scenarios in [21].

Despite these advances, the integration of movable antenna techniques into IRS-assisted systems remains largely underexplored. In particular, enabling the movement of IRS elements introduces new design opportunities but also brings fundamental challenges. First, the position optimization of IRS elements is strongly coupled with transmit beamforming and phase shift design, leading to a highly non-convex and high-dimensional optimization problem. Second, the movement of IRS elements incurs additional power consumption, which has been largely overlooked in existing studies that mainly focus on rate maximization. As a result, whether the performance gain brought by element mobility can compensate for the increased energy cost remains an open problem.

To address the above issues, this paper investigates the secrecy energy efficiency (SEE) maximization problem in a downlink multiple-input single-output (MISO) system assisted by an ME-IRS. By jointly optimizing the transmit beamforming, IRS phase shifts, and element positions, we aim to fully exploit the spatial degrees of freedom introduced by element mobility while explicitly accounting for the power consumed by the associated movement. The main contributions of this paper are summarized as follows:

- We investigate an ME-IRS-assisted secure communication system and formulate an SEE maximization problem by jointly optimizing the transmit beamforming, IRS phase shifts, and element positions. In contrast to what has been presented in existing works, the proposed formulation explicitly incorporates the power consumption induced by element movement, leading to a more practical and comprehensive system design.
- To solve the resulting highly non-convex and coupled problem, we develop an alternating optimization (AO)-based framework. Specifically, the original problem is decomposed into three subproblems, which are efficiently addressed by jointly leveraging Dinkelbach’s method for fractional programming, semidefinite relaxation (SDR), and successive convex approximation (SCA) across different subproblems. The proposed algorithm is guaranteed to converge to a stationary solution with polynomial-time computational complexity.
- Numerical results demonstrate that the proposed ME-IRS significantly outperforms conventional IRS configurations in terms of SEE. In particular, the performance gain becomes more pronounced as the number of elements and the movable region size increase, gradually becoming saturated beyond a certain range. This reveals that a properly designed movable region can effectively balance performance improvement and implementation cost.

Notations: In this paper, boldface capital letters, boldface lowercase letters, and regular lowercase letters are adopted to represent matrices, vectors, and scalars, respectively. The set of real values, the set of non-negative real values, and the set of complex values are denoted by R, $R^{+}$, and C, respectively. $j=\sqrt{-1}$ represents the imaginary unit, and $C^{M\times N}$ represents a complex space with a dimension of M×N. The transpose and Hermitian transpose of a matrix are denoted by ${\left(\cdot \right)}^{T}$ and ${\left(\cdot \right)}^{H}$, respectively. $\left|\cdot \right|$, $∥\cdot ∥$, and ${∥\cdot ∥}_{F}$ denote the absolute value of a complex scalar, the $l_{2}$-norm of a vector, and the *F*-norm of a matrix, respectively. For a given $X\in C^{M\times N}$, X⪰0 means that X is a positive semidefinite matrix. diag(X) and Diag(x) represent a column vector with its entries extracted from the diagonal elements of X and a diagonal matrix with x being the diagonal elements, respectively. The trace and rank of X are denoted by Tr(X) and rank(X), respectively. For a continuous differentiable function f(X), $\nabla_{X}f\left(\cdot \right)$ denotes the gradient of f(·) with respect to X. $\lambda_{\max}\left(X\right)$ and $\lambda_{\min}\left(X\right)$ denote the maximum and minimum eigenvalues of X, respectively. $I_{N}$ denotes an N×N identity matrix.

## 2. System Model

**Figure 1:**
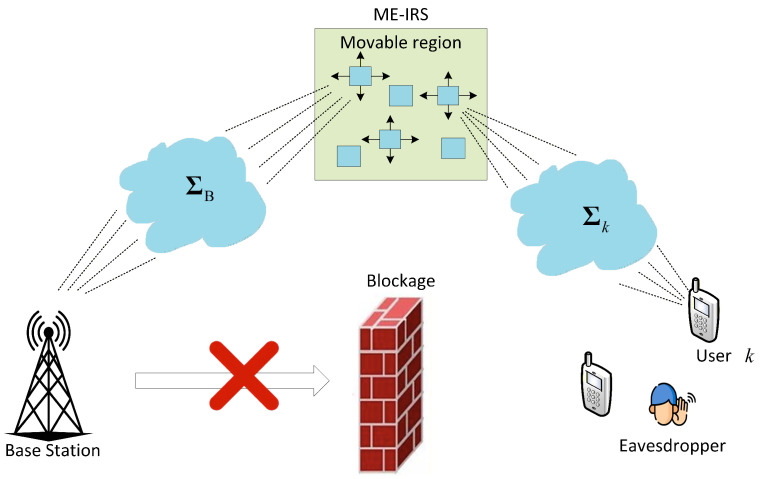
The considered ME-IRS-enabled secure communication system.

We consider a downlink MISO wireless communication system, as illustrated in Figure 1, where a base station (BS) equipped with *M* fixed-position antennas (FPAs) serves *K* users, indexed by k∈K={1,2,⋯,K}, each equipped with a single FPA. Meanwhile, a passive eavesdropper is present in the system attempting to wiretap the information intended for legitimate users. To enhance the security performance of the system, an intelligent reflecting surface comprising *N* movable elements is deployed to assist transmission. The direct link between the BS and the users is assumed to be blocked due to severe obstacles in the propagation environment, which is a commonly adopted assumption in IRS-related literature [5,17]. Without loss of generality, the FPAs at the BS and the MEs at the IRS are described in their respective local coordinate systems. The coordinate of the *m*th FPA is denoted by $b_{m}={\left[x_{m},y_{m}\right]}^{T}$, while the coordinate of the *n*th ME is denoted by $p_{n}={\left[x_{n},y_{n}\right]}^{T}\in C_{I}$, where $C_{I}$ denotes the movable region at the IRS.

### 2.1. Channel Model

Since the movable region for the ME-IRS is significantly smaller than both the propagation distance between the BS and the IRS and that between the IRS and the users, the far-field condition generally holds for BS–IRS and IRS–user channels. Specifically, the movement of IRS elements does not affect the angle of departure (AoD), angle of arrival (AoA), or channel gain of the involved channels. All communication channels are assumed to be quasi-static during the considered time period. To evaluate the upper bound of performance enabled by the ME-IRS, we assume perfect channel state information (CSI) of both legitimate users and the eavesdropper at the BS. (The CSI, including AoDs, AoAs, and complex coefficients of the multipath components, can be jointly estimated using compressed sensing techniques; details can be found in [22,23]. The extension to imperfect CSI is left as a possible direction for future work.)

Adopting the field response channel model proposed in [14], we assume that the channel between the BS and the ME-IRS comprises $L_{B}$ paths. The field response vector (FRV) of the *n*th reflecting element for the BS–IRS link is denoted by

(1) $$\begin{array}{c} g\left(b_{m}\right)={\left[e^{j\frac{2\pi }{\lambda }b_{m}^{T}\rho_{B,1}},\cdots ,e^{j\frac{2\pi }{\lambda }b_{m}^{T}\rho_{B,L_{B}}}\right]}^{T}, \end{array}$$

(2) $$\begin{array}{c} f\left(p_{n}\right)={\left[e^{j\frac{2\pi }{\lambda }p_{n}^{T}\rho_{I,1}},\cdots ,e^{j\frac{2\pi }{\lambda }p_{n}^{T}\rho_{I,L_{B}}}\right]}^{T}, \end{array}$$

where $\rho_{B/I,l}={\left[\cos\varphi_{B/I,l}\sin\phi_{B/I,l},\sin\varphi_{B/I,l}\right]}^{T}$, with $\varphi_{B/I,l}$ and $\phi_{B/I,l}$ representing the elevation and azimuth AoA/AoD of the *l*th path, respectively, and λ denotes the carrier wavelength. Note that $\left\{g\left(b_{m}\right),m\in \left[1,M\right]\right\}$ are constant vectors owing to the fixed position of the antennas at the BS. The channel response of the BS–IRS link is thus given by

(3) $$H\left(P\right)={\left(F\left(P\right)\right)}^{H}\Sigma_{B}G,$$

where $F\left(P\right)=[f\left(p_{1}\right),\cdots ,f\left(p_{N}\right)]$, $G=[g\left(b_{1}\right),\cdots ,g\left(b_{M}\right)]$, $P=[p_{1},\cdots ,p_{N}]$. $\Sigma_{B}\in C^{L_{B}\times L_{B}}$ represents the path response matrix (PRM) of the BS–IRS link.

Similarly, the FRV at the *n*th ME for the user *k* and the eavesdropper can be obtained by

(4) $$\begin{array}{c} u_{k}\left(p_{n}\right)={\left[e^{j\frac{2\pi }{\lambda }p_{n}^{T}\rho_{k,1}},\cdots ,e^{j\frac{2\pi }{\lambda }p_{n}^{T}\rho_{k,L_{k}}}\right]}^{T}, \end{array}$$

(5) $$\begin{array}{c} e\left(p_{n}\right)={\left[e^{j\frac{2\pi }{\lambda }p_{n}^{T}\rho_{e,1}},\cdots ,e^{j\frac{2\pi }{\lambda }p_{n}^{T}\rho_{e,L_{e}}}\right]}^{T}, \end{array}$$

respectively, where $\rho_{k/e,l}={\left[\cos\varphi_{k/e,l}\sin\phi_{k/e,l},\sin\varphi_{k/e,l}\right]}^{T}$ with $\varphi_{k/e,l}$ and $\phi_{k/e,l}$ representing the elevation and azimuth AoA of the *l*th path for user *k*/eavesdropper, respectively. Consequently, the channel response between the IRS and user *k*/eavesdropper are thus given by

(6) $$v_{k}\left(P\right)=1_{L_{k}}^{H}\Sigma_{k}U_{k}\left(P\right),$$

(7) $$v_{e}\left(P\right)=1_{L_{e}}^{H}\Sigma_{e}U_{e}\left(P\right),$$,

respectively, where $U_{k}\left(P\right)=\left[u_{k}\left(p_{1}\right),\cdots ,u_{k}\left(p_{N}\right)\right]$, $U_{e}\left(P\right)=\left[u_{e}\left(p_{1}\right),\cdots ,u_{e}\left(p_{N}\right)\right]$. $1_{L}\in C^{L\times 1}$ denotes an all-ones vector. $\Sigma_{k/e}\in C^{L_{k/e}\times L_{k/e}}$ represents the PRM of IRS–user/eavesdropper link. Consequently, the cascade channel from BS to user *k* and eavesdropper can be expressed as

(8) $$\begin{array}{c} h_{k}\left(P,\Theta \right)=v_{k}\left(P\right)\Theta H\left(P\right), \end{array}$$

(9) $$\begin{array}{c} h_{e}\left(P,\Theta \right)=v_{e}\left(P\right)\Theta H\left(P\right), \end{array}$$

respectively, where

(10) $$\Theta =\mathrm{Diag}(\left[\theta_{1},\cdots ,\theta_{N}\right])$$

denotes the phase shift matrix at the IRS.

From (8) and (9), it can be observed that the cascaded channels of both the legitimate users and the eavesdropper are jointly determined by the IRS position matrix P and the phase shift matrix Θ. This reveals that, by adaptively optimizing P and Θ, the system can enhance the legitimate channels while suppressing the eavesdropping channels, thereby improving secrecy performance.

### 2.2. Secrecy Energy Efficiency

According to the above channel model, the signal received by the *k*th user and the eavesdropper can be expressed as

(11) $$y_{k}=h_{k}\left(P,\Theta \right)\sum_{\bar{k}\in K}w_{\bar{k}}s_{\bar{k}}+n_{k},$$

and

(12) $$y_{e}=h_{e}\left(P,\Theta \right)\sum_{\bar{k}\in K}w_{\bar{k}}s_{\bar{k}}+n_{e},$$

respectively, in which $s_{k}\in C$ represents the information-bearing signal for the *k*th user; $w_{k}\in C^{M\times 1}$ is the corresponding beamforming vector; and $n_{k/e}∼\mathrm{CN}\left(0,\sigma_{k/e}^{2}\right)$ represents the additive white Gaussian noise (AWGN), with $\sigma_{k/e}^{2}$ being the corresponding noise variance. Without loss of generality, we assume $E\{s_{k}^{H}s_{k}\}=1$ and $E\{s_{k}^{H}s_{k^{\prime }}\}=0$ for $\left\{k\neq k^{\prime }\right\}$. The achievable rate for user *k* is thus given by

(13) $$R_{k}=\log_{2}\left(1+\frac{{\left|h_{k}\left(P,\Theta \right)w_{k}\right|}^{2}}{\sum_{\bar{k}\in K\setminus k}{\left|h_{k}\left(P,\Theta \right)w_{\bar{k}}\right|}^{2}+\sigma_{k}^{2}}\right).$$

As for the eavesdropper, the achievable eavesdropping rate to decode the information signal of user *k* is denoted as

(14) $$R_{e,k}=\log_{2}\left(1+\frac{{\left|h_{e}\left(P,\Theta \right)w_{k}\right|}^{2}}{\sum_{\bar{k}\in K\setminus k}{\left|h_{e}\left(P,\Theta \right)w_{\bar{k}}\right|}^{2}+\sigma_{e}^{2}}\right).$$

Then, the secrecy rate for user *k* is defined as

(15) $$R_{S,k}={\left[R_{k}-R_{e,k}\right]}^{+},$$

where ${\left[\cdot \right]}^{+}=\max\left(0,\cdot \right)$.

It is worth noting that although the movement of IRS elements introduces an additional degree of freedom for improving the system’s secrecy performance, it also incurs extra power consumption compared with conventional fixed-position IRS. In this paper, we adopt an affine power model [24], and the total power consumption is defined as

(16) $$P_{\mathrm{tot}}=\zeta \sum_{k\in K}{∥w_{k}∥}^{2}+NP_{\mathrm{move}}+P_{S},$$

where ζ>1 denotes the reciprocal of the power amplifier efficiency at the BS, while $P_{\mathrm{move}}$ represents the circuit power consumption associated with the movement of each IRS element. (In this paper, for analytical tractability, the movement power is modeled as a constant term that captures the average energy consumption associated with element movement. Extending the model to incorporate more realistic movement dynamics would be an interesting direction for future work.) $P_{S}$ accounts for the static hardware power consumption at both the BS and the IRS. Accordingly, the secrecy energy efficiency (SEE) is defined as

(17) $$\eta =\frac{\sum_{k\in K}R_{S,k}}{P_{\mathrm{tot}}}.$$

### 2.3. Problem Formulation

In this paper, our objective is to maximize the SEE η by jointly optimizing the transmitting beamformers $w_{k}$, the phase shift matrix Θ and the position matrix P at the IRS. The resulting optimization problem can be formulated as follows:

(18) $$\begin{array}{cc} \; & \max_{w_{k},p_{n},\Theta }\;\;\eta \\ s.t.\; & C1:\;p_{n}\in C_{I},\;\forall n,\\ \; & C2:\;∥p_{i}-p_{j}∥\;\geq D,\forall i\neq j,\\ \; & C3:\;\sum_{k\in K}{∥w_{k}∥}^{2}\leq P_{\max},\\ \; & C4:\;|\Theta (n,n)|=1,\;\forall n\in [1,N],\\ \; & C5:\;R_{S,k}\geq R_{k,0},\forall k\in K. \end{array}$$

In problem (18), constraint C1 ensures that the IRS elements remain within the movable region. Constraint C2 is imposed to avoid collisions among the elements, where *D* denotes the specified minimum distance between adjacent elements. Constraint C3 represents the constraint on total transmit power at the BS, where $P_{\max}$ denotes the corresponding power budget. Constraint C4 specifies the unit-modulus constraint for each IRS element. Constraint C5 represents the minimum rate requirement of each legitimate user, where $R_{k,0}\geq 0$ denotes the corresponding secrecy rate threshold.

## 3. Alternating Optimization

**Figure 2:**
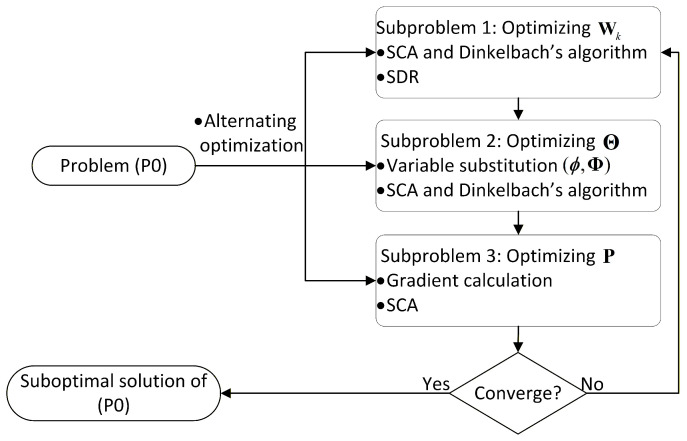
Flow chart of the proposed AO algorithm.

The optimization problem formulated in (18) is intractable owing to the fractional-form objective function, the strong coupling among the optimization variables, and the non-convex constraints. To address this issue, we adopt an alternating optimization (AO) scheme and decompose problem (18) into three subproblems. The overall algorithm is shown in Figure 2. Specifically, in Subproblem 1, the beamforming vectors $w_{k}$ are optimized with the phase shift matrix Θ fixed. In Subproblem 2, the phase shift matrix Θ is optimized with P fixed. In Subproblem 3, the position matrix P is optimized for a given $w_{k}$ and Θ.

### 3.1. Beamforming Design

For a given Θ and P, the problem in (18) can be equivalently expressed as

(19) $$\begin{array}{cc} \; & \max_{W_{k},S_{k}}\;\;\frac{\sum_{k\in K}S_{k}}{\zeta \sum_{k\in K}\mathrm{Tr}\left(W_{k}\right)+c_{w}}\;\;\\ s.t.\; & C3:\;\sum_{k\in K}\mathrm{Tr}\left(W_{k}\right)\leq P_{\max},\\ \; & C5:\;S_{k}\geq R_{k,0},\forall k\in K,\\ \; & C6:\;W_{k}⪰0,\forall k\in K,\\ \; & C7:\;\mathrm{rank}(W_{k})=1,\forall k\in K,\\ \; & C8:\;R_{S,k}\geq S_{k},\forall k\in K, \end{array}$$

where $W_{k}=w_{k}w_{k}^{H}$, while $\left\{S_{k},k\in K\right\}$ are introduced auxiliary variables. Constraints C6 and C7 are imposed to ensure that $w_{k}$ can be recovered from $W_{k}$. At this stage, the optimization problem in (19) remains non-convex owing to the fractional-form objective function, the rank constraint C7, and the rate constraint C8. To address these issues, we first rewrite $R_{S,k}$ as

(20) $$\begin{array}{cc} R_{S,k}= & \log_{2}\left(\frac{\sum_{\bar{k}\in K}{\left|h_{k}w_{\bar{k}}\right|}^{2}+\sigma_{k}^{2}}{\sum_{\bar{k}\in K\setminus k}{\left|h_{k}w_{\bar{k}}\right|}^{2}+\sigma_{k}^{2}}\right)-\log_{2}\left(\frac{\sum_{\bar{k}\in K}{\left|h_{e}w_{\bar{k}}\right|}^{2}+\sigma_{e}^{2}}{\sum_{\bar{k}\in K\setminus k}{\left|h_{e}w_{\bar{k}}\right|}^{2}+\sigma_{e}^{2}}\right)\\ = & \underset{Q_{1}\left(W\right)}{\underset{︸}{\left(\log_{2}\left(\sum_{\bar{k}\in K}\mathrm{Tr}\left(M_{k}W_{\bar{k}}\right)+\sigma_{k}^{2}\right)+\log_{2}\left(\sum_{\bar{k}\in K\setminus k}\mathrm{Tr}\left(M_{e}W_{\bar{k}}\right)+\sigma_{e}^{2}\right)\right)}}\\ \; & -\underset{Q_{2}\left(W\right)}{\underset{︸}{\left(\log_{2}\left(\sum_{\bar{k}\in K\setminus k}\mathrm{Tr}\left(M_{k}W_{\bar{k}}\right)+\sigma_{k}^{2}\right)+\log_{2}\left(\sum_{\bar{k}\in K}\mathrm{Tr}\left(M_{e}W_{\bar{k}}\right)+\sigma_{e}^{2}\right)\right)}}, \end{array}$$

where $M_{k}=h_{k}h_{k}^{H}$, $M_{e}=h_{e}h_{e}^{H}$, and $W={\left\{W_{k}\right\}}_{k\in K}$. Note that although the expression of $R_{S,k}$ is non-convex, it can be expressed as the difference of two concave functions, i.e., $Q_{1}$ and $Q_{2}$, which can be convexified by employing the successive convex approximation (SCA) technique [25]. Specifically, by leveraging the first-order Taylor expansion of $Q_{2}$, for a feasible solution $W^{\left(t\right)}={\left\{W_{k}^{\left(t\right)}\right\}}_{k\in K}$, where *t* denotes the iteration index, a lower bound of $R_{S,k}$ can be obtained as

(21) $$\begin{array}{cc} R_{S,k}= & Q_{1}\left(W\right)-Q_{2}\left(W\right)\\ \geq & Q_{1}\left(W\right)-Q_{2}\left(W^{\left(t\right)}\right)-\sum_{\bar{k}\in K}\mathrm{Tr}\left(\nabla_{W_{\bar{k}}}^{H}Q_{2}\left(W^{\left(t\right)}\right)\left(W_{\bar{k}}-W_{\bar{k}}^{\left(t\right)}\right)\right)={\bar{R}}_{S,k}, \end{array}$$

where

(22) $$\nabla_{W_{\bar{k}}}^{H}Q_{2}\left(W\right)=\left\{\begin{array}{c} \frac{M_{e}/\ln2}{\sum_{\bar{k}\in K}\mathrm{Tr}\left(M_{e}W_{\bar{k}}\right)+\sigma_{e}^{2}},\;\bar{k}=k,\\ \frac{M_{k}/\ln2}{\sum_{\bar{k}\in K\setminus k}\mathrm{Tr}\left(M_{k}W_{\bar{k}}\right)+\sigma_{k}^{2}}+\frac{M_{e}/\ln2}{\sum_{\bar{k}\in K}\mathrm{Tr}\left(M_{e}W_{\bar{k}}\right)+\sigma_{e}^{2}},\;\bar{k}\neq k. \end{array}\right.$$

After that, a lower bound of problem (19) can be obtained by solving the following problem:

(23) $$\begin{array}{cc} \; & \max_{W_{k},S_{k}}\;\;\frac{\sum_{k\in K}S_{k}}{\zeta \sum_{k\in K}\mathrm{Tr}\left(W_{k}\right)+c_{w}}\;\;\\ s.t.\; & C3,\;C5,\;C6,\;C7,\\ \; & \bar{C8}:\;{\bar{R}}_{S,k}\geq S_{k},\forall k\in K. \end{array}$$

Generally, the optimization problem in (23) can be classified as a fractional programming problem, which can be optimally solved by leveraging Dinkelbach’s method [26]. Specifically, the optimal solution of the problem in (23) can be obtained by iteratively solving the following problem:

(24) $$\begin{array}{cc} \; & \max_{W_{k},S_{k}}\;\;\sum_{k\in K}S_{k}-\eta^{t_{w}}\left(\zeta \sum_{k\in K}\mathrm{Tr}\left(W_{k}\right)+c_{w}\right)\;\;\\ s.t.\; & C3,\;C5,\;C6,\;C7,\;\bar{C8}, \end{array}$$

where

(25) $$\eta^{t_{w}}=\frac{\sum_{k\in K}S_{k}^{\left(t_{w}-1\right)}}{\zeta \sum_{k\in K}\mathrm{Tr}\left(W_{k}^{\left(t_{w}-1\right)}\right)+c_{w}}$$

and $t_{w}$ denotes the iteration index of Dinkelbach’s method. Now, the only non-convexity of the problem in (24) arises from the rank constraint C7. To address this issue, we adopt the semidefinite relaxation (SDR) technique by dropping the rank constraint C7. As a result, the problem becomes convex with respect to all optimization variables and can therefore be efficiently solved by standard convex optimization solvers such as CVX [27].

Note that although the SDR technique is applied to relax the rank constraint C7, it can be shown through the following theorem that a rank-one solution can always be obtained or constructed from the optimal solution.

**Theorem** **1.** *If the version of problem* (24) *with a relaxed rank constraint is feasible, then either the optimal solution $W^{*}$ satisfies rank 1 or we can reconstruct a rank-1 solution as ${\bar{W}}^{*}$, which can achieve the same optimal value of $W_{k}^{*}$.*

**Proof.**  Please refer to Appendix A.    □

Moreover, the optimality and convergence of Dinkelbach’s method have been well established in the literature. Therefore, an optimal solution to problem (23) can be obtained by iteratively solving problem (24), which serves as a lower bound to the problem (19). Furthermore, by iteratively solving problem (23), the performance of the obtained suboptimal solution can be gradually improved. The overall SCA-based algorithm for solving problem (19) is summarized in Algorithm 1.
**Algorithm 1** SCA-based beamforming design**Initialize:** the convergence tolerance μ→0, the maximum number of iterations $t_{\max}$,        the iteration index $t=t_{w}=1$, $W^{\left(t\right)}$, $\eta^{\left(t_{w}\right)}=\eta_{\mathrm{out}}^{\left(t\right)}=0$;
  1:**repeat**$\left\{\mathrm{SCA}\mathrm{loop}\right\}$  2:      **repeat**$\left\{{\mathrm{Dinkelbach}}^{\prime }s\mathrm{method}\right\}$  3:            Solve the SDR version of problem (24) for given $W^{\left(t\right)}$ to obtain the optimized $W^{*}$;  4:            **Set** $t_{w}=t_{w}+1$, and $\eta^{\left(t_{w}\right)}=\frac{\sum_{k\in K}S_{k}^{\left(t_{w}-1\right)}}{\zeta \sum_{k\in K}\mathrm{Tr}\left(W_{k}^{\left(t_{w}-1\right)}\right)+c_{w}}$;  5:      **until** $\frac{|\eta^{\left(t_{w}\right)}-\eta^{\left(t_{w}-1\right)}|}{|\eta^{\left(t_{w}\right)}|}\leq \mu$ or $t_{w}=t_{\max}$;  6:      **Set** $t_{w}=1$, t=t+1, $W^{\left(t\right)}=W^{*}$, $\eta_{\mathrm{out}}^{\left(t\right)}=\eta^{\left(t_{w}\right)}$;  7:**until** $\frac{|\eta_{\mathrm{out}}^{\left(t\right)}-\eta_{\mathrm{out}}^{\left(t-1\right)}|}{|\eta_{\mathrm{out}}^{\left(t\right)}|}\leq \mu$ or $t=t_{\max}$


### 3.2. Phase Shift Design

With fixed beamforming vectors $w_{k}$ and element position matrix P, problem (18) can be equivalently expressed as

(26) $$\begin{array}{cc} \; & \max_{\Theta ,S_{k}}\;\;\sum_{k\in K}S_{k}\\ s.t. & C4:\;|\Theta (n,n)|=1,\;\forall n\in [1,N],\\ \; & C5:\;S_{k}\geq R_{k,0},\forall k\in K,\\ \; & C8:\;R_{S,k}\geq S_{k},\forall k\in K. \end{array}$$

First, we rewrite the cascaded channel response from the BS to user *k* and the eavesdropper as

(27) $$\begin{array}{cc} \; & h_{k}\left(\Theta \right)=v_{k}\Theta H={\mathrm{diag}}^{H}\left(\Theta \right)\mathrm{Diag}\left(v_{k}\right)H, \end{array}$$

(28) $$\begin{array}{cc} \; & h_{e}\left(\Theta \right)=v_{e}\Theta H={\mathrm{diag}}^{H}\left(\Theta \right)\mathrm{Diag}\left(v_{e}\right)H, \end{array}$$

respectively. Then, the secrecy rate can be recast as

(29) $$R_{S,k}=\log_{2}\left(1+\frac{\mathrm{Tr}(\Phi B_{k,k})}{\sum_{\bar{k}\in K\setminus k}\mathrm{Tr}\left(\Phi B_{\bar{k},k}\right)+\sigma_{k}^{2}}\right)-\log_{2}\left(1+\frac{\mathrm{Tr}(\Phi B_{k,e})}{\sum_{\bar{k}\in K\setminus k}\mathrm{Tr}\left(\Phi B_{\bar{k},e}\right)+\sigma_{e}^{2}}\right),$$

where θ=diag(Θ), $\Phi =\theta \theta^{H}$,

(30) $$\begin{array}{cc} \; & B_{\bar{k},k}=\mathrm{Diag}\left(v_{k}\right)Hw_{\bar{k}}w_{\bar{k}}^{H}H^{H}{\mathrm{Diag}}^{H}\left(v_{k}\right),\;\forall \bar{k},k\in K, \end{array}$$

(31) $$\begin{array}{cc} \; & B_{\bar{k},e}=\mathrm{Diag}\left(v_{e}\right)Hw_{\bar{k}}w_{\bar{k}}^{H}H^{H}{\mathrm{Diag}}^{H}\left(v_{e}\right),\forall \bar{k}\in K. \end{array}$$

Then, $R_{S,k}$ can be further rewritten as

(32) $$\begin{array}{cc} R_{S,k}= & \underset{J_{1}\left(\Phi \right)}{\underset{︸}{\log_{2}\left(\sum_{\bar{k}\in K}\mathrm{Tr}\left(\Phi B_{\bar{k},k}\right)+\sigma_{k}^{2}\right)+\log_{2}\left(\sum_{\bar{k}\in K\setminus k}\mathrm{Tr}\left(\Phi B_{\bar{k},e}\right)+\sigma_{e}^{2}\right)}}\\ \; & -\underset{J_{2}\left(\Phi \right)}{\underset{︸}{\left(\log_{2}\left(\sum_{\bar{k}\in K\setminus k}\mathrm{Tr}\left(\Phi B_{\bar{k},k}\right)+\sigma_{k}^{2}\right)+\log_{2}\left(\sum_{\bar{k}\in K}\mathrm{Tr}\left(\Phi B_{\bar{k},e}\right)+\sigma_{e}^{2}\right)\right)}}, \end{array}$$

which is in the form of the difference of two concave functions. As in Section 3.1, we adopt the SCA technique to obtain a lower bound for $R_{S,k}$. Specifically, for a given feasible solution set $\Phi^{\left(t\right)}$, where *t* denotes the iteration index, a lower bound of $R_{S,k}$ can be formulated as

(33) $$R_{S,k}\geq {\hat{R}}_{S,k}=J_{1}\left(\Phi \right)-J_{2}\left(\Phi^{\left(t\right)}\right)-\nabla_{\Phi }^{H}J_{2}\left(\Phi^{\left(t\right)}\right)\left(\Phi -\Phi^{\left(t\right)}\right),$$

where

(34) $$\nabla_{\Phi }^{H}J_{2}\left(\Phi \right)=\frac{\sum_{\bar{k}\in K\setminus k}B_{\bar{k},k}/\ln2}{\sum_{\bar{k}\in K\setminus k}\mathrm{Tr}\left(\Phi B_{\bar{k},k}\right)+\sigma_{k}^{2}}+\frac{\sum_{\bar{k}\in K}B_{\bar{k},e}/\ln2}{\sum_{\bar{k}\in K}\mathrm{Tr}\left(\Phi B_{\bar{k},e}\right)+\sigma_{e}^{2}}.$$

Thus, a lower bound for problem (26) can be obtained by solving the following problem:

(35) $$\begin{array}{cc} \; & \max_{\Phi ,S_{k}}\;\;\sum_{k\in K}S_{k}\\ s.t.\; & \bar{C4}:\;\Phi \left(n,n\right)=1,\;\forall n\in \left[1,N\right],\\ \; & C5:\;S_{k}\geq R_{k,0},\forall k\in K,\\ \; & C8:\;{\hat{R}}_{S,k}\geq S_{k},\forall k\in K,\\ \; & C9:\;\Phi ⪰0,\\ \; & C10:\;\mathrm{rank}(\Phi )=1, \end{array}$$

in which constraints C9 and C10 are imposed to ensure that θ can be recovered from Φ. The optimization problem (35) remains non-convex owing to the rank constraint C10. To tackle this issue, we reformulate the rank constraint C10 as

(36) $$\bar{C10}:{∥\Phi ∥}_{*}-{∥\Phi ∥}_{2}\leq 0.$$

Subsequently, by employing the SCA technique, a convex subset of $\bar{C10}$ at any feasible point $\Phi^{\left(t\right)}$ can be expressed as

(37) $$\tilde{C10}:{∥\Phi ∥}_{*}-{∥\Phi^{\left(t\right)}∥}_{2}-\mathrm{Tr}\left(\lambda_{\max}\left(\Phi^{\left(t\right)}\right)\lambda_{\max}^{H}\left(\Phi^{\left(t\right)}\right)\left(\Phi -\Phi^{\left(t\right)}\right)\right)\leq 0.$$

Consequently, the optimization problem is reformulated as

(38) $$\begin{array}{cc} \; & \max_{\Phi ,S_{k}}\;\;\sum_{k\in K}S_{k}\\ s.t.\; & \bar{C4},\;C5,\;C8,\;C9,\;\tilde{C10} \end{array}$$

Note that problem (38) is jointly convex with respect to all optimization variables and can therefore be efficiently solved by standard convex optimization solvers such as CVX. Furthermore, the obtained lower bound of problem (26) can be progressively improved by iteratively solving problem (38).

### 3.3. Element Position Optimization

By fixing the beamforming vectors $w_{k}$ and phase shift matrix Θ, the optimization problem (18) can be equivalently reformulated as

(39) $$\begin{array}{cc} \; & \max_{p_{n},S_{k}}\;\;\sum_{k\in K}S_{k}\\ s.t.\; & C1:\;p_{n}\in C_{I},\;\forall n,\\ \; & C2:\;∥p_{i}-p_{j}∥\geq D,\forall i\neq j,\\ \; & C5:\;S_{k}\geq R_{k,0},\forall k\in K,\\ \; & C8:\;R_{S,k}\geq S_{k},\forall k\in K. \end{array}$$

To begin, we recast the cascaded channel response $h_{k}\left(P\right)$ as

(40) $$\begin{array}{cc} h_{k}\left(P\right) & =\theta^{H}\mathrm{Diag}\left(v_{k}\left(P\right)\right)H\left(P\right)\\ \; & =\theta^{H}\mathrm{Diag}\left(1_{L_{k}}^{H}\Sigma_{k}U_{k}\left(P\right)\right){\left(F\left(P\right)\right)}^{H}\Sigma_{B}G\\ \; & =\sum_{n=1}^{N}\theta_{n}1_{L_{k}}^{H}\Sigma_{k}u_{k}\left(p_{n}\right){\left(f(p_{n})\right)}^{H}\Sigma_{B}G\\ \; & =\sum_{n=1}^{N}r_{n,k}\Upsilon_{k}\left(p_{n}\right)\Sigma_{B}G, \end{array}$$

where $r_{n,k}=\theta_{n}1_{L_{k}}^{H}\Sigma_{k}$ and $\Upsilon_{k}\left(p_{n}\right)=u_{k}\left(p_{n}\right){\left(f(p_{n})\right)}^{H}$. Similarly, $h_{e}\left(P\right)$ can be reformulated as

(41) $$h_{e}\left(P\right)=\sum_{n=1}^{N}r_{n,e}\Upsilon_{e}\left(p_{n}\right)\Sigma_{B}G,$$

where $r_{n,e}=\theta_{n}1_{L_{e}}^{H}\Sigma_{e}$ and $\Upsilon_{e}\left(p_{n}\right)=e\left(p_{n}\right){\left(f(p_{n})\right)}^{H}$. Subsequently, the secrecy rate of user *k* can be formulated as

(42) $$\begin{array}{cc} R_{S,k}= & \log_{2}\left(1+\frac{|\sum_{n=1}^{N}r_{n,k}\Upsilon_{k}\left(p_{n}\right)t_{k}{|}^{2}}{\sum_{\bar{k}\in K\setminus k}{\left|\sum_{n=1}^{N}r_{n,k}\Upsilon_{k}\left(p_{n}\right)t_{\bar{k}}\right|}^{2}+\sigma_{k}^{2}}\right)\\ \; & \;\;-\log_{2}\left(1+\frac{|\sum_{n=1}^{N}r_{n,e}\Upsilon_{e}\left(p_{n}\right)t_{k}{|}^{2}}{\sum_{\bar{k}\in K\setminus k}{\left|\sum_{n=1}^{N}r_{n,e}\Upsilon_{e}\left(p_{n}\right)t_{\bar{k}}\right|}^{2}+\sigma_{k}^{2}}\right), \end{array}$$

where $t_{k}=\Sigma_{B}Gw_{k}$.

Accordingly, the optimization problem (39) remains non-convex owing to the strong coupling among the position vectors of all elements. To address this issue, we adopt an iterative scheme in which the position of one element, $p_{n}$, is optimized in each iteration while the positions of the remaining elements, $\left\{p_{\bar{n}},\bar{n}\neq n\right\}$, are kept fixed. Subsequently, the SCA technique is employed to handle the non-convex constraint C8 utilizing the second-order Taylor expansion of $R_{S,k}$. Specifically, for a given feasible point $p_{n}^{\left(t\right)}$, $R_{S,k}\left(p_{n}\right)$ can be approximated as

(43) $$R_{S,k}\left(p_{n}\right)\approx R_{S,k}\left(p_{n}^{\left(t\right)}\right)+\nabla R_{S,k}\left(p_{n}^{\left(t\right)}\right)\left(p_{n}-p_{n}^{\left(t\right)}\right)+\frac{1}{2}{\left(p_{n}-p_{n}^{\left(t\right)}\right)}^{T}\nabla^{2}R_{S,k}\left(p_{n}^{\left(t\right)}\right)\left(p_{n}-p_{n}^{\left(t\right)}\right),$$

where $\nabla R_{S,k}\left(p_{n}\right)\in R^{2}$ and $\nabla^{2}R_{S,k}\left(p_{n}\right)\in R^{2\times 2}$ represent the gradient vector and Hessian matrix of $R_{S,k}\left(p_{n}\right)$, respectively. The detailed derivation of $\nabla R_{S,k}\left(p_{n}\right)$ and $\nabla^{2}R_{S,k}\left(p_{n}\right)$ is provided in Appendix B. Then, by constructing a positive number $\chi_{n,k}$ that satisfies $\chi_{n,k}I⪰\nabla^{2}R_{S,k}\left(p_{n}\right)$, a lower bound of $R_{S,k}\left(p_{n}\right)$ can be obtained as

(44) $$R_{S,k}\left(p_{n}\right)\geq {\bar{R}}_{S,k}\left(p_{n}\right)=R_{S,k}\left(p_{n}^{\left(t\right)}\right)+\nabla R_{S,k}\left(p_{n}^{\left(t\right)}\right)\left(p_{n}-p_{n}^{\left(t\right)}\right)+\frac{\chi_{n,k}}{2}{\left(p_{n}-p_{n}^{\left(t\right)}\right)}^{T}\left(p_{n}-p_{n}^{\left(t\right)}\right),$$

in which $\chi_{n,k}$ is set as $\chi_{n,k}=\;{∥\nabla^{2}R_{S,k}\left(p_{n}\right)∥}_{F}\geq {∥\nabla^{2}R_{S,k}\left(p_{n}\right)∥}_{2}$; therefore, $\chi_{n,k}I⪰\nabla^{2}R_{S,k}\left(p_{n}\right)$.

Similarly, the non-convex constraint C2 can be convexified via the SCA technique based on its first-order Taylor expansion, which can be expressed as

(45) $$\bar{C2}:\frac{1}{∥p_{n}^{\left(t\right)}-p_{\bar{n}}∥}{\left(p_{n}^{\left(t\right)}-p_{\bar{n}}\right)}^{T}\left(p_{n}-p_{\bar{n}}\right)\geq D,1\leq \bar{n}\leq N,\bar{n}\neq n.$$

Consequently, a lower bound for problem (39) can be obtained by iteratively solving the following problem:

(46) $$\begin{array}{cc} \; & \max_{p_{n},S_{k}}\;\;\sum_{k\in K}S_{k}\\ s.t.\; & C1,\;\bar{C2},\;C5,\\ \; & \bar{C8}:\;{\bar{R}}_{S,k}\geq S_{k},\forall k\in K. \end{array}$$

Problem (46) is a quadratic programming problem and can thus be efficiently solved by standard convex optimization solvers such as CVX.

### 3.4. Overall Algorithm and Computational Complexity

By alternating optimizing the beamforming vectors $w_{k}$, the phase shift matrix Θ, and the element position matrix P, the achieved SEE η forms a non-decreasing sequence,

(47) $$\begin{array}{cc} \eta^{\left(i\right)} & =\eta \left(w_{k}^{\left(i\right)},\Theta^{\left(i\right)},P^{\left(i\right)}\right)\\ \; & \leq \eta \left(w_{k}^{\left(i+1\right)},\Theta^{\left(i\right)},P^{\left(i\right)}\right)\\ \; & \leq \eta \left(w_{k}^{\left(i+1\right)},\Theta^{\left(i+1\right)},P^{\left(i\right)}\right)\\ \; & \leq \eta \left(w_{k}^{\left(i+1\right)},\Theta^{\left(i+1\right)},P^{\left(i+1\right)}\right)=\eta^{\left(i+1\right)}, \end{array}$$

where *i* represents the iteration index of the AO iterations. Moreover, owing to the transmit power constraint at the BS, the SEE has an upper bound, which guarantees the convergence of the proposed AO algorithm.

In addition, the computational complexities of the three subproblems are summarized as follows:

- Problem (24): The number of decision variables is on the order of $n=KM^{2}$. The problem involves *K* linear matrix inequality (LMI) constraints of size *M* and 2K+1 LMI constraints of size 1. When a standard interior-point method (IPM) [28] is employed, the computational complexity is $O\;\left(I_{out}I_{D}\sqrt{m}\left(KM^{3}n+KM^{2}n^{2}+n^{3}\right)\right)$, where m=KM+2K+1, while $I_{D}$ and $I_{out}$, respectively, denote the numbers of iterations required for the Dinkelbach method and the outer SCA loop to converge [29].
- Problem (38): Similarly, the complexity for solving problem (38) using the IPM is $O\;(I_{2}\sqrt{m}\left(N^{3}n+N^{2}n^{2}+n^{3}\right))$, where $n=N^{2}$ and m=2K+2N+1.
- Problem (46): The computational complexities for calculating $r_{n,k}$, $t_{k}$, $a_{n}^{k,\bar{k}}$, $\nabla R_{S,k}\left(p_{n}\right)$, and $\chi_{n,k}$ are $O(\sum_{k}L_{k})$, $O(ML_{B})$, $O(NKL_{B}\sum_{k}L_{k})$, $O(L_{B}\sum_{k}L_{k})$, and $O(L_{B}\sum_{k}L_{k})$, respectively. In addition, the complexity for solving problem (46) using the IPM is $O(N^{1.5})$. Therefore, the computational complexity of Algorithm 2 is $O\;(NI_{\mathrm{SCA}}\left(N^{1.5}+NKL_{B}\sum_{k}L_{k}\right))$, where $I_{\mathrm{SCA}}$ denotes the number of iterations required for the SCA algorithm to converge.

Therefore, the overall AO algorithm exhibits polynomial-time computational complexity, which makes it suitable for practical implementation.
**Algorithm 2** SCA-based element position optimization algorithm**Initialize:** the convergence tolerance μ→0, the maximum number of iterations $t_{\max}$,        ${\left\{p_{n}^{\left(1\right)}\right\}}_{n=1}^{N}$;
  1:**for** n=1→N **do**  2:      **Set** SCA index t=1;  3:      **repeat** $\left\{\mathrm{SCA}\mathrm{loop}\right\}$  4:            Solve the problem (46) for given $p_{n}^{\left(t\right)}$ to obtain the optimized $p_{n}^{*}$;  5:            **Set** $R_{\mathrm{sum}}^{\left(t\right)}=\sum_{k\in K}S_{k}^{\left(t\right)}$, and t=t+1;  6:      **until** $\frac{|R_{\mathrm{sum}}^{\left(t\right)}-R_{\mathrm{sum}}^{\left(t-1\right)}|}{|R_{\mathrm{sum}}^{\left(t\right)}|}\leq \mu$ or $t=t_{\max}$;  7:**end for**  8:**Output:** ${\left\{p_{n}\right\}}_{n=1}^{N}$.


## 4. Simulation Results

In this section, simulation results are presented to evaluate the secrecy performance improvement achieved by the proposed ME-IRS and to verify the effectiveness of the proposed AO algorithm. A three-dimensional Cartesian coordinate system is considered, where the BS is located at (0, 0, 0) m and the IRS is deployed at (10, 10, 5) m. The legitimate users and the eavesdropper are uniformly distributed within a circular region centered at (25, 0, 0) m with a radius of 10 m. The AoAs and AoDs are uniformly distributed in [−π/2,π/2]. A geometric channel model is adopted, where the transmit and receive paths exhibit a one-to-one correspondence, i.e., $\Sigma_{k}$, $\Sigma_{B}$, and $\Sigma_{e}$ are diagonal matrices. The diagonal entries are modeled as $\beta =\beta_{0}d^{-\alpha_{0}}$, where $\beta_{0}=-30$ dB denotes the channel power gain at the reference distance of 1 m, while $\alpha_{0}=2.2$ represents the path loss exponents [30]. Unless otherwise specified, the system parameters are set as in Table 1.

Moreover, the following baseline schemes are considered for performance comparison:

- Fixed-position IRS (FP-IRS): The positions of the IRS elements are fixed and uniformly distributed within the region C.
- Random-position IRS (RP-IRS): The positions of the IRS elements are randomly generated within the region C and remain fixed during the optimization. The beamforming vectors and the phase shift matrix are jointly optimized using the proposed algorithm.
- Discrete-position IRS (DP-IRS): The movable region is quantized into a set of candidate grid points with uniform spacing of λ/2, and a greedy algorithm is employed to determine the position of each element. Note that this scheme is functionally similar to the dense candidate grid with a subset activation/switching approach.

Note that for all three baseline schemes, the beamforming vectors and the phase shift matrix are jointly optimized using the proposed algorithm.

### 4.1. Convergence Performance

**Figure 3:**
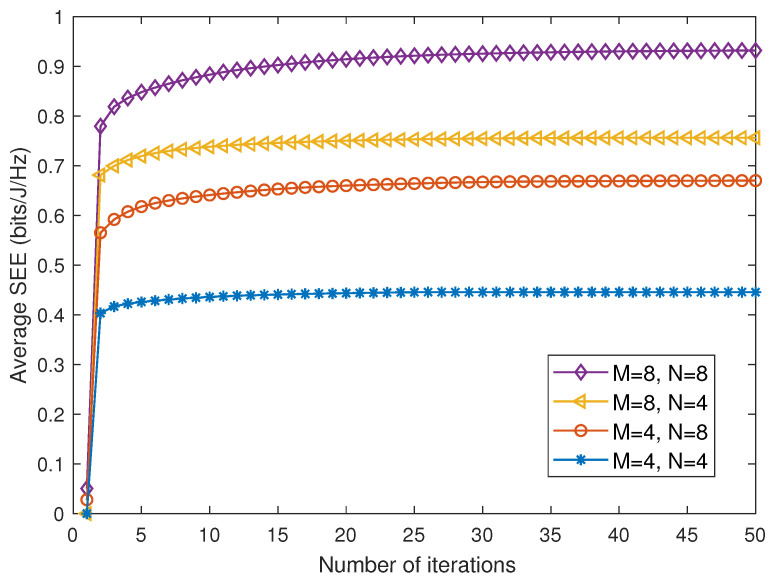
Convergence performance of the proposed AO algorithm.

Figure 3 illustrates the convergence behavior of the proposed AO algorithm. It can be observed that the achieved SEE increases monotonically with the number of iterations and converges within about 20 iterations. This result confirms the effectiveness and stability of the proposed AO framework, which is consistent with the convergence analysis in Section 3.4. Moreover, increasing the numbers of antennas *M* and IRS elements *N* improves the secrecy performance but requires more iterations to converge. This is because larger *M* and *N* provide additional degrees of freedom for beamforming and phase shift design, while also enlarging the solution space and increasing the optimization complexity.

### 4.2. SEE Versus Transmit Power Budget

**Figure 4:**
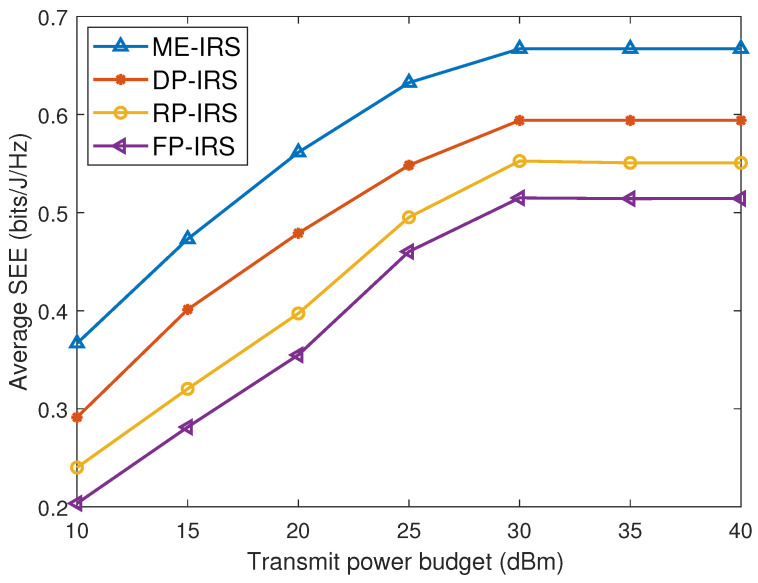
Average SEE versus the transmit power budget.

Figure 4 illustrates the SEE performance versus the maximum transmit power $P_{\max}$ for different IRS configurations. It can be observed that the proposed ME-IRS scheme consistently achieves the highest SEE compared with the baseline schemes. In addition, the SEE increases with the transmit power and gradually approaches saturation as $P_{\max}$ becomes large. This is because increasing the transmit power improves the achievable secrecy rate, while the power consumption increases simultaneously, leading to a diminishing SEE gain at high transmit power levels. Moreover, compared with the conventional FP-IRS, the proposed ME-IRS achieves approximately a 30% SEE improvement. This performance gain mainly stems from the additional degrees of freedom introduced by the position optimization of IRS elements. Furthermore, even the DP-IRS can achieve noticeable performance improvement over the FP-IRS, demonstrating the effectiveness of position optimization for IRS elements.

### 4.3. SEE Under Different Number of IRS Elements

**Figure 5:**
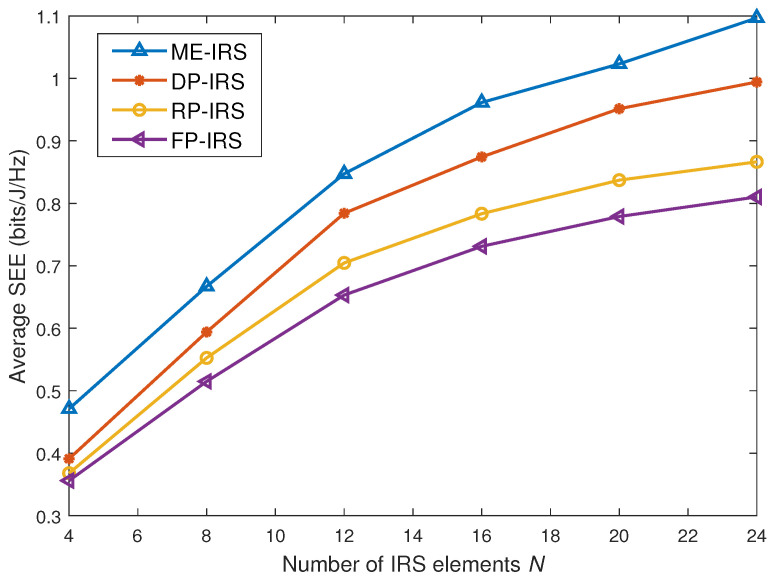
Average SEE versus the number of IRS elements.

Figure 5 depicts the SEE performance versus the number of IRS elements *N* under different IRS configurations. It can be observed that the performance gain achieved by the proposed ME-IRS becomes more pronounced as *N* increases. In particular, compared with the conventional FP-IRS, the SEE improvement conferred by the ME-IRS enlarges as *N* increases. This is because the movable IRS elements provide additional spatial degrees of freedom, which can be better exploited when more elements are available for position optimization.

### 4.4. SEE Versus Circuit Power Consumption of ME

**Figure 6:**
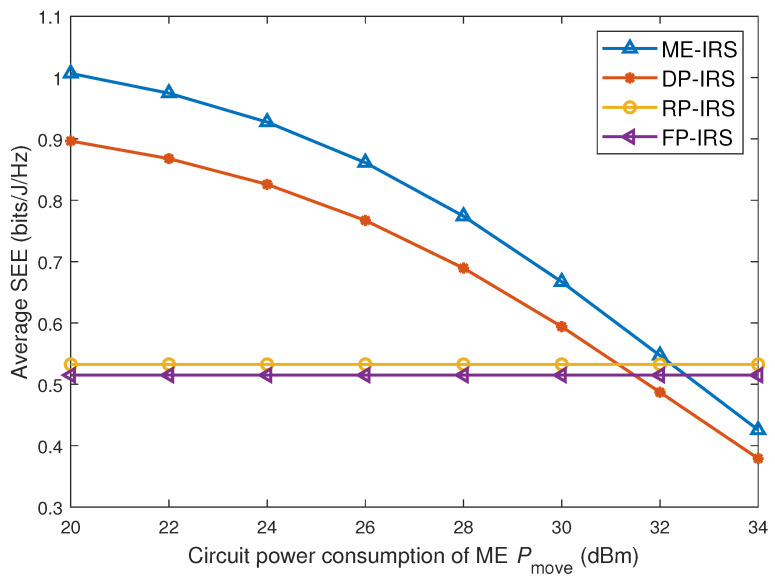
Average SEE versus circuit power consumption of ME $P_{\mathrm{move}}$.

Figure 6 illustrates the SEE performance of different IRS schemes under varying IRS element movement power. It can be observed that when the IRS movement power $P_{\mathrm{move}}$ is low, the performance improvement from position optimization significantly enhances the system SEE, mainly benefiting from the additional spatial degrees of freedom provided by the movement of IRS elements. As $P_{\mathrm{move}}$ increases, the proportion of extra power consumption in the total system power gradually rises. Although position optimization can still improve the secrecy rate, the corresponding gain in SEE gradually diminishes. When $P_{\mathrm{move}}$ becomes sufficiently high ($P_{\mathrm{move}}\geq 32$ dBm), the secrecy rate gain from position optimization can no longer compensate for the movement power, resulting in an SEE even lower than that of the fixed-position IRS scheme. Therefore, in practical deployments, the IRS movement power should be carefully controlled to balance performance and power consumption. Moreover, a hybrid scheme combining fixed-position and movable IRSs can be adopted to reduce power consumption while still leveraging the benefits of position optimization.

### 4.5. SEE Under Different Movable Region Sizes

**Figure 7:**
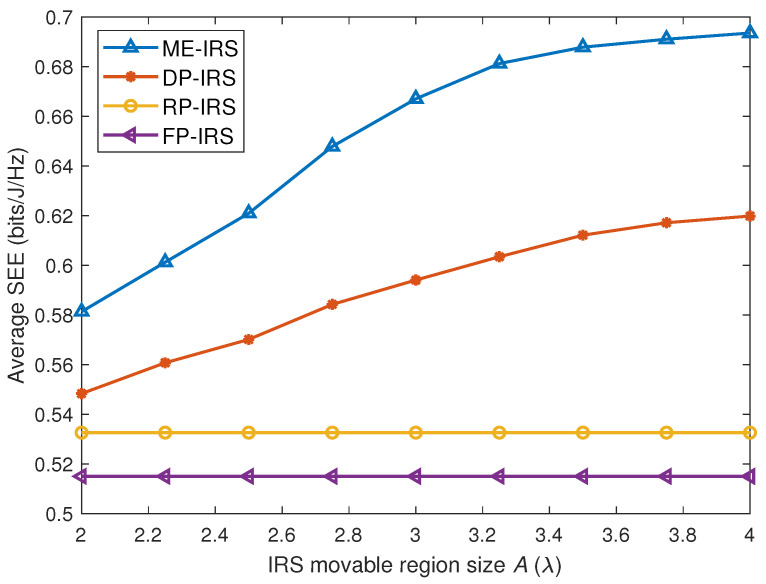
Average SEE versus size of movable region *A*.

Figure 7 evaluates the SEE under different movable region sizes. As the movable region enlarges, the SEE achieved by the proposed ME-IRS improves owing to the increased flexibility for element position optimization. The DP-IRS configuration also provides certain performance gains compared with the FP-IRS, since selecting positions from discrete grids still introduces additional spatial flexibility. However, as the movable region becomes larger, the performance gap between the ME-IRS and the DP-IRS gradually increases. This is because the continuous position optimization in the ME-IRS can better exploit the enlarged feasible region, whereas the DP-IRS is restricted by the predefined discrete grids.

In addition, the SEE gain gradually becomes saturated when the movable region becomes sufficiently large, since the available spatial degrees of freedom are already well exploited. This reveals that the movable region size should be properly selected, as an excessively large region may increase implementation complexity without bringing significant performance improvement.

## 5. Conclusions

In this paper, we investigated an ME-IRS-assisted secure communication system and studied the SEE maximization problem by jointly optimizing the transmit beamforming, IRS phase shifts, and element positions. To tackle the resulting highly coupled and non-convex problem, an efficient AO framework was developed by integrating Dinkelbach’s method, SDR, and SCA. The proposed algorithm was shown to converge with polynomial-time computational complexity. Simulation results demonstrated that the proposed ME-IRS significantly outperforms conventional IRS configurations in terms of SEE. In particular, the mobility of IRS elements provides additional spatial degrees of freedom, leading to notable performance gains. In addition, the results reveal that enlarging the movable region improves the SEE by providing greater flexibility for position optimization, while the performance gain gradually becomes saturated when the region becomes sufficiently large, suggesting that an appropriate movable region size should be carefully selected in practical deployments.

## Figures and Tables

**Table 1:** System Parameters.

Parameter	Value
System carrier center frequency	2.4 GHz
Number of BS antennas *M*	4
Number of IRS element *N*	8
Number of users *K*	2
Number of paths $L_{B}=L_{k}=L_{e}$	5
Noise power	−96 dBm
Static hardware power consumption $P_{S}$	40 dBm
Circuit power consumption of ME $P_{\mathrm{move}}$	30 dBm
Transmit power budget $P_{\max}$	30 dBm
Minimum distance between elements *D*	λ/2
Movable region C=A×A	A=3λ
Power amplifier inefficiency factor ζ	1/0.3

## Data Availability

The original contributions presented in this study are included in the article. Further inquiries can be directed to the corresponding author.

## Appendix A. Proof of Theorem 1

First, we rewrite problem (24) in its epigraph form as

(A1) $$\begin{array}{cc} \; & \min_{W_{k},S_{k}}\;\;\eta^{t_{w}}\left(\zeta \sum_{k\in K}\mathrm{Tr}\left(W_{k}\right)+c_{w}\right)-\sum_{k\in K}S_{k}\;\;\\ s.t.\; & C3,\;C5,\;C6,\;\\ \; & \bar{C8}:\log_{2}\left(\alpha_{k}+\sigma_{k}^{2}\right)+\log_{2}\left(\beta_{k}+\sigma_{e}^{2}\right)-Q_{2}\left(W^{\left(t\right)}\right)\\ & \;\;-\sum_{\bar{k}\in K}\mathrm{Tr}\left(\nabla_{W_{\bar{k}}}^{H}Q_{2}\left(W^{\left(t\right)}\right)\left(W_{\bar{k}}-W_{\bar{k}}^{\left(t\right)}\right)\right)\geq S_{k},\;\forall k\in K,\\ \; & C11:\sum_{\bar{k}\in K}\mathrm{Tr}\left(M_{k}W_{\bar{k}}\right)\geq \alpha_{k},\;\forall k\in K, \end{array}$$

(A2) $$\begin{array}{cc} \; & C12:\sum_{\bar{k}\in K\setminus k}\mathrm{Tr}\left(M_{e}W_{\bar{k}}\right)\geq \beta_{k},\;\forall k\in K. \end{array}$$

Then, the Lagrangian function in terms of $W_{k}$ is given by

(A3) $$\begin{array}{cc} L= & \eta^{t_{w}}\zeta \sum_{k\in K}\mathrm{Tr}\left(W_{k}\right)+\gamma \sum_{k\in K}\mathrm{Tr}\left(W_{k}\right)-\sum_{k\in K}\mathrm{Tr}\left(\Gamma_{k}W_{k}\right)+\sum_{k\in K}\vartheta_{k}\sum_{\bar{k}\in K}\mathrm{Tr}\left(\nabla_{W_{\bar{k}}}^{H}Q_{2}\left(W^{\left(t\right)}\right)W_{\bar{k}}\right)\\ \; & -\sum_{k\in K}\iota_{k}\sum_{\bar{k}\in K}\mathrm{Tr}\left(M_{k}W_{\bar{k}}\right)-\sum_{k\in K}\kappa_{k}\sum_{\bar{k}\in K\setminus k}\mathrm{Tr}\left(M_{e}W_{\bar{k}}\right)+c, \end{array}$$

where *c* involves all terms independent of $W_{k}$. γ, $\Gamma_{k}$, $\vartheta_{k}$, $\iota_{k}$, and $\kappa_{k}$ denote the Lagrangian multipliers associated with constraints C3, C6, $\bar{C8}$, C11, and C12, respectively. Correspondingly, the dual problem is given by

(A4) $$\begin{array}{cc} \; & \max_{\Xi }\;g\left(\Theta \right),\\ s.t.\; & \gamma \geq 0,\Gamma_{k}⪰0,\vartheta_{k}\geq 0,\iota_{k}\geq 0,\kappa_{k}\geq 0,\;\forall k\in K, \end{array}$$

where Ξ represents the collection of all Lagrangian multipliers and $g\left(\Xi \right)=\min_{W_{k}}L\left(W_{k},\Xi \right)$ is the dual function. Since problem (A1) is jointly convex with respect to the optimization variables and satisfies the Slater constraint qualification [31], strong duality holds and the optimal solution of problem (A1) can be obtained by solving the following problem:

(A5) $$\;g\left(\Xi^{*}\right)=\min_{W_{k}}\sum_{k\in K}\mathrm{Tr}\left(\Psi_{k}\left(\Xi^{*}\right)W_{k}\right)+c,$$

where $\Psi_{k}\left(\Xi \right)$ involves all the terms associated with $W_{k}$ and is a function of all Lagrangian multipliers, while $\Xi^{*}$ represents the optimal solution to the dual problem. Since problem (24) is feasible and the duality gap is zero, $\Psi_{k}\left(\Xi^{*}\right)$ must be positive semidefinite, i.e., $\Psi_{k}\left(\Xi^{*}\right)⪰0$, to ensure that the optimal Lagrangian dual function is not unbounded [32]. Next, we assume that $W_{k}^{*}$ is the optimal solution of problem (A5) with rank *A*. Then, $W_{k}^{*}$ can be expressed as

(A6) $$W_{k}^{*}=\sum_{a=1}^{A}\lambda_{a,k}p_{a,k}p_{a,k}^{H},$$

where $\lambda_{a,k}$ and $p_{a,k}$ represent the *a*th non-zero eigenvalue and the corresponding eigenvector of $W_{k}$, respectively. Then, we construct another feasible solution for problem (A5) as

(A7) $$\begin{array}{c} \sum_{k\in K}\mathrm{Tr}\left(\Psi_{k}\left(\Theta^{*}\right){\bar{W}}_{k}^{*}\right)\overset{\left(b\right)}{\leq }\sum_{k\in K}\mathrm{Tr}\left(\Psi_{k}\left(\Theta^{*}\right)W_{k}^{*}\right), \end{array}$$

where the equality in (b) holds if and only if A=1 or $\mathrm{Tr}\left(\Psi_{k}\left(\Theta^{*}\right)W_{k}^{*}\right)=0$, for any *k*. Furthermore, to keep the optimality of $W_{k}^{*}$, the equality in (b) must hold. Thus, we have $\mathrm{rank}\left(W_{k}^{*}\right)=1$ or we can recover a rank-1 solution from $W_{k}^{*}$ as

(A8) $${\bar{W}}_{k}^{*}=\lambda_{a,k}p_{a,k}p_{a,k}^{H},\forall a\in \left[1,A\right],$$

which not only achieves the same objective value but also satisfies the constraint of being a rank-1 matrix.

## Appendix B. Derivation of ∇*R*_S,*k*_(p_*n*_) and ∇^2^ *R*_S,*k*_(p_*n*_)

For the sake of simplicity of expression, we first define

(A9) $$f_{k,\bar{k}}\left(p_{n}\right)=r_{n,k}\Upsilon_{k}\left(p_{n}\right)t_{\bar{k}},\;\;\forall k,\bar{k}\in K.$$

By leveraging the chain rule, $\nabla R_{S,k}\left(p_{n}\right)$ can be expressed as

(A10) $$\nabla R_{S,k}\left(p_{n}\right)=\frac{\partial R_{k}}{\partial p_{n}}-\frac{\partial R_{e,k}}{\partial p_{n}},$$

where

(A11) $$\frac{\partial R_{k}}{\partial p_{n}}=\frac{\frac{\partial f_{k,k}}{\partial p_{n}}\left(\sum_{\bar{k}\in K\setminus k}f_{k,\bar{k}}+\sigma_{k}^{2}\right)-f_{k,k}\sum_{\bar{k}\in K\setminus k}\frac{\partial f_{k,\bar{k}}}{\partial p_{n}}}{\ln2\left(\sum_{\bar{k}\in K}f_{k,\bar{k}}+\sigma_{k}^{2}\right)\left(\sum_{\bar{k}\in K\setminus k}f_{k,\bar{k}}+\sigma_{k}^{2}\right)}.$$

Subsequently, we further define

(A12) $$a_{n}^{k,\bar{k}}=\left|a_{n}^{k,\bar{k}}\right|e^{j∠a_{n}^{k,\bar{k}}}=\sum_{\bar{n}\neq n}^{N}r_{\bar{n},k}\Upsilon_{k}\left(p_{\bar{n}}\right)t_{\bar{k}}$$

where $|a_{n}^{k,\bar{k}}|$ and $∠a_{n}^{k,\bar{k}}$ denote the amplitude and phase of $a_{n}^{k,\bar{k}}$, respectively. Moreover, let $r_{n,k}^{i}=\left|r_{n,k}^{i}\right|e^{j∠r_{n,k}^{i}}$ and $t_{k}^{l}=\left|t_{k}^{l}\right|e^{j∠t_{k}^{l}}$ denote the *i*th element of $r_{n,k}$ and the *l*th element of $t_{k}$, respectively. Then, $f_{k,\bar{k}}$ can be rewritten as

(A13) $$f_{k,\bar{k}}={\left|\sum_{i=1}^{L_{k}}\sum_{l=1}^{L_{B}}|r_{n,k}^{i}\left|\right|t_{\bar{k}}^{l}|e^{j\frac{2\pi }{\lambda }\left(p_{n}^{T}\rho_{k,i}-p_{n}^{T}\rho_{I,l}+∠r_{n,k}^{i}+∠t_{\bar{k}}^{l}\right)}+\left|a_{n}^{k,\bar{k}}\right|e^{j∠a_{n}^{k,\bar{k}}}\right|}^{2}.$$

The partial derivatives of $f_{k,\bar{k}}$ w.r.t. $x_{n}$ and $y_{n}$ are thus given by

(A14) $$\frac{\partial f_{k,\bar{k}}}{\partial x_{n}}=\frac{4\pi |a_{n}^{k,\bar{k}}|}{\lambda }\sum_{i=1}^{L_{k}}\sum_{l=1}^{L_{B}}\mu_{k}^{i,l}|r_{n,k}^{i}\left|\right|t_{\bar{k}}^{l}|\sin\left(∠a_{n}^{k,\bar{k}}-\delta_{k,\bar{k}}^{i,l}\left(p_{n}\right)\right),$$

(A15) $$\frac{\partial f_{k,\bar{k}}}{\partial y_{n}}=\frac{4\pi |a_{n}^{k,\bar{k}}|}{\lambda }\sum_{i=1}^{L_{k}}\sum_{l=1}^{L_{B}}\nu_{k}^{i,l}|r_{n,k}^{i}\left|\right|t_{\bar{k}}^{l}|\sin\left(∠a_{n}^{k,\bar{k}}-\delta_{k,\bar{k}}^{i,l}\left(p_{n}\right)\right),$$

where

(A16) $$\begin{array}{cc} \; & \delta_{k,\bar{k}}^{i,l}\left(p_{n}\right)=\frac{2\pi }{\lambda }\left(\mu^{i,l}x_{n}+\nu^{i,l}y_{n}\right)+∠r_{n,k}^{i}+∠t_{\bar{k}}^{l}, \end{array}$$

(A17) $$\begin{array}{cc} \; & \mu_{k}^{i,l}=\cos\varphi_{k,i}\sin\phi_{k,i}-\cos\varphi_{I,l}\sin\phi_{I,l}, \end{array}$$

(A18) $$\begin{array}{cc} \; & \nu_{k}^{i,l}=\sin\varphi_{k,l}-\sin\varphi_{I,l}. \end{array}$$

Then, the derivative of $R_{k}$ can be obtained by substituting (A14) and (A15) into (A11). The derivative of $R_{e,k}$ can be derived in a similar manner and is omitted for brevity.

Regarding the Hessian matrix $\nabla^{2}R_{S,k}\left(p_{n}\right)$,

(A19) $$\nabla^{2}R_{S,k}\left(p_{n}\right)=\left[\begin{array}{cc} \frac{\partial^{2}R_{k}}{\partial x_{n}^{2}}-\frac{\partial^{2}R_{e,k}}{\partial x_{n}^{2}} & \frac{\partial^{2}R_{k}}{\partial x_{n}\partial y_{n}}-\frac{\partial^{2}R_{e,k}}{\partial x_{n}\partial y_{n}}\\ \frac{\partial^{2}R_{k}}{\partial y_{n}\partial x_{n}}-\frac{\partial^{2}R_{e,k}}{\partial y_{n}\partial x_{n}} & \frac{\partial^{2}R_{k}}{\partial y_{n}^{2}}-\frac{\partial^{2}R_{e,k}}{\partial y_{n}^{2}} \end{array}\right].$$

On the basis of (A11), we can derive

(A20) $$\begin{array}{cc} \frac{\partial^{2}R_{k}}{\partial x_{n}^{2}}= & \frac{\sum_{\bar{k}\in K}\frac{\partial f_{k,\bar{k}}}{\partial x_{n}}\left(\sum_{\bar{k}\in K\setminus k}f_{k,\bar{k}}+\sigma_{k}^{2}\right)+\sum_{\bar{k}\in K\setminus k}\frac{\partial f_{k,\bar{k}}}{\partial x_{n}}\left(\sum_{\bar{k}\in K}f_{k,\bar{k}}+\sigma_{k}^{2}\right)}{\ln2{\left(\sum_{\bar{k}\in K}f_{k,\bar{k}}+\sigma_{k}^{2}\right)}^{2}{\left(\sum_{\bar{k}\in K\setminus k}f_{k,\bar{k}}+\sigma_{k}^{2}\right)}^{2}}\left(\frac{\partial f_{k,k}}{\partial x_{n}}\left(\sum_{\bar{k}\in K\setminus k}f_{k,\bar{k}}+\sigma_{k}^{2}\right)-f_{k,k}\sum_{\bar{k}\in K\setminus k}\frac{\partial f_{k,\bar{k}}}{\partial x_{n}}\right)\\ \; & +\frac{\frac{\partial^{2}f_{k,k}}{\partial x_{n}^{2}}\left(\sum_{\bar{k}\in K\setminus k}f_{k,\bar{k}}+\sigma_{k}^{2}\right)-f_{k,k}\sum_{\bar{k}\in K\setminus k}\frac{\partial^{2}f_{k,\bar{k}}}{\partial x_{n}^{2}}}{\ln2\left(\sum_{\bar{k}\in K}f_{k,\bar{k}}+\sigma_{k}^{2}\right)\left(\sum_{\bar{k}\in K\setminus k}f_{k,\bar{k}}+\sigma_{k}^{2}\right)}. \end{array}$$

Similarly, $\frac{\partial^{2}R_{k}}{\partial x_{n}\partial y_{n}}$, $\frac{\partial^{2}R_{k}}{\partial y_{n}\partial x_{n}}$, and $\frac{\partial^{2}R_{k}}{\partial y_{n}^{2}}$ can be expressed in terms of $\frac{\partial^{2}f_{k,\bar{k}}}{\partial x_{n}^{2}}$, $\frac{\partial^{2}f_{k,\bar{k}}}{\partial x_{n}\partial y_{n}}$, $\frac{\partial^{2}f_{k,\bar{k}}}{\partial y_{n}\partial x_{n}}$, and $\frac{\partial^{2}f_{k,\bar{k}}}{\partial y_{n}^{2}}$, where

(A21) $$\begin{array}{cc} \; & \frac{\partial^{2}f_{k,\bar{k}}}{\partial x_{n}^{2}}=-\frac{8\pi^{2}\left|a_{n}^{k,\bar{k}}\right|}{\lambda^{2}}\sum_{i=1}^{L_{k}}\sum_{l=1}^{L_{B}}{\left(\mu_{k}^{i,l}\right)}^{2}|r_{n,k}^{i}\left|\right|t_{\bar{k}}^{l}|\sin\left(∠a_{n}^{k,\bar{k}}-\delta_{k,\bar{k}}^{i,l}\left(p_{n}\right)\right), \end{array}$$

(A22) $$\begin{array}{cc} \; & \frac{\partial^{2}f_{k,\bar{k}}}{\partial x_{n}\partial y_{n}}=\frac{\partial^{2}f_{k,\bar{k}}}{\partial y_{n}\partial x_{n}}=\frac{8\pi^{2}\left|a_{n}^{k,\bar{k}}\right|}{\lambda^{2}}\sum_{i=1}^{L_{k}}\sum_{l=1}^{L_{B}}\mu_{k}^{i,l}\nu_{k}^{i,l}|r_{n,k}^{i}\left|\right|t_{\bar{k}}^{l}|\sin\left(∠a_{n}^{k,\bar{k}}-\delta_{k,\bar{k}}^{i,l}\left(p_{n}\right)\right), \end{array}$$

(A23) $$\begin{array}{cc} \; & \frac{\partial^{2}f_{k,\bar{k}}}{\partial y_{n}^{2}}=-\frac{8\pi^{2}\left|a_{n}^{k,\bar{k}}\right|}{\lambda^{2}}\sum_{i=1}^{L_{k}}\sum_{l=1}^{L_{B}}{\left(\nu_{k}^{i,l}\right)}^{2}|r_{n,k}^{i}\left|\right|t_{\bar{k}}^{l}|\sin\left(∠a_{n}^{k,\bar{k}}-\delta_{k,\bar{k}}^{i,l}\left(p_{n}\right)\right). \end{array}$$

Furthermore, $\nabla^{2}R_{e,k}$ can be obtained following the same procedure, and therefore, $\nabla^{2}R_{S,k}$ can be derived accordingly.
